# Food and beverage marketing in primary and secondary schools in Canada

**DOI:** 10.1186/s12889-019-6441-x

**Published:** 2019-01-28

**Authors:** Monique Potvin Kent, Cayley E. Velazquez, Elise Pauzé, Olivia Cheng-Boivin, Noami Berfeld

**Affiliations:** 1 Faculty of Medicine, School of Epidemiology and Public Health, University of Ottawa, 600 Peter Morand Cres., Room 301J, Ottawa, Ontario K1G5Z3 Canada; 2 Faculty of Science and Horticulture, 12666 72 Ave, Surrey, British Columbia V3W 2M8 Canada; 3 Faculty of Medicine, School of Medicine, University of Ottawa, 451 Smyth Road, Ottawa, Ontario K1H 8M5 Canada; 4 Faculty of Health Sciences, Interdisciplinary School of Health Sciences, University of Ottawa, 25 University Private, Ottawa, Ontario K1N 7K4 Canada

**Keywords:** Food marketing, Schools, Canada, Policy, Food environment, Obesity, Self-regulation, Children, Adolescents

## Abstract

**Background:**

Unhealthy food marketing is considered a contributor to childhood obesity. In Canada, food marketing in schools is mostly self-regulated by industry though it is sometimes restricted through provincial school policies. The purpose of this study was to document the type of food marketing activities occurring in Canadian schools and examine differences by school characteristics.

**Methods:**

An online survey was sent to public primary and secondary schools from 27 school boards in Ontario, British Columbia, and Nova Scotia and was completed by 154 Principals in spring 2016. This survey queried the type of food marketing occurring in schools including advertisements, food product displays, fundraising, exclusive marketing agreements, and incentive programs, among others. The occurrence of food marketing was described using frequencies, medians, and ranges. Chi-square and Fisher Exact tests were conducted to assess school-level differences in the frequency of marketing activities by school type (primary versus secondary), province (Ontario versus British Columbia), and the socio-economic status of most students (low versus middle/high income). The significance level was set at α < 0.05 for all tests.

**Results:**

Overall, 84% of schools reported at least one type of food marketing and the median number of distinct types of marketing per school was 1 (range 0–6). The most frequently reported forms of marketing were the sale of branded food, particularly chocolate, pizza, and other fast food, for fundraising (64% of schools); food advertisements on school property (26%), and participation in incentive programs (18%). Primary schools (*n* = 108) were more likely to report participating in incentive programs (25%) and selling branded food items (72%) compared to secondary schools (*n* = 46; 2 and 43% respectively; *p* < 0.01). Conversely, secondary schools were more likely to report food advertising on school property (56%), exclusive marketing arrangements with food companies (43%), and food product displays (19%) than primary schools (13, 5 and 2%, respectively; *p* < 0.01).

**Conclusion:**

The presence of food marketing in most participating schools suggests that the current patchwork of policies that restrict food marketing in Canadian schools is inadequate. Comprehensive restrictions should be mandated by government in both primary and secondary schools to protect children and youth from this marketing.

## Background

According to recent estimates, the prevalence of obesity among children and adolescents went from 11 to 124 million globally between 1975 and 2016 [1]. In Canada, rates of childhood obesity have doubled over the last 30 years [2], and almost one third of children and adolescents aged 5–17 have excess weight or obesity [3]. The frequency of food and beverage marketing (hereafter referred to as food marketing) directed at children and youth has also increased in tandem with rising obesity rates, not only in traditional media such as television, but also in new media such as the Internet and smartphones and in schools and other places where youth gather [4]. Food marketing, much of which promotes unhealthy products [5], has been shown to directly impact children’s food preferences, food requests, and short-term food intake [4–6]. Moreover, food marketing has been associated with childhood obesity [4], a risk factor in the development of chronic diseases such as type 2 diabetes and heart disease [7].

Marketing is defined as “any form of commercial communication or message that is designed to, or has the effect of, increasing the recognition, appeal and/or consumption of particular products and services.” [8] It includes any activity that “acts to advertise or otherwise promote a product or service” [8]. Product, price, place (i.e. how products are made accessible to a specific group of consumers) and promotion (i.e. persuasive communication approaches such as advertising used to transmit information to consumers) are often cited as the four tools of marketing [9]. Schools are an attractive setting for food companies interested in influencing children’s dietary behaviors. They offer an opportunity to advertise (and sell) directly to a captive audience of young people segmented by age, among whom fostered brand loyalty may lead to lifelong consumption of promoted products [10, 11]. Schools are also “uncluttered marketing environments” [12] in which children spend more than 180 days a year [13]. This allows for prolonged exposure to food marketing. Many different forms of marketing have been identified in schools to date. These include: (1) appropriation of space (i.e., using school property to promote companies through naming rights or advertising), (2) corporate sponsorship of programs, events or contests (i.e., corporations paying for or subsidizing activities in return for recognition), (3) exclusive marketing arrangements (i.e., contractual agreements giving companies exclusive rights to sell their products on school property), (4) incentive programs (i.e., programs that provide a commercial reward in return for student achievement), (5) sponsored educational materials (i.e., curricular materials provided to schools by corporate companies), (6) electronic marketing (i.e., the use of television, Internet or other media to promote brands) and (7) fundraising (i.e., direct sales of services or products of which a portion of the revenues are given to schools to raise money) [10, 14].

Food marketing in schools has been documented extensively in the United States (US) and has been found to be widespread and detrimental to the school nutrition environment [15–19]. Studies have also shown that the extent and nature of school-based marketing may vary between primary and secondary schools and between schools attended by students of lower and higher socio-economic status [14, 17, 18, 20, 21]. In Canada, much like other high-income countries and those that are less economically developed, considerably less is known about the presence of food marketing in schools [14, 16, 20, 22]. According to a national survey on commercialism in Canadian primary and secondary schools conducted in 2004–05, 27% of schools overall had an exclusive marketing agreement with two large beverage companies, and in-school advertisements for both companies were the most predominant compared to other companies [14]. More recently, Velazquez et al. [20] documented considerable presence of promotional materials for unhealthy foods and beverages in schools in Vancouver, British Columbia despite school board policies that prohibit such advertising. Many Canadian provinces have introduced policies restricting the sale of certain food items based on nutrition standards [23–25]; however, none include comprehensive restrictions of food marketing according to an analysis of publicly available school policy documents (Chambers S and Potvin Kent M, unpublished). This was also found to be the case for most public school boards in British Columbia, Ontario, and Nova-Scotia (Chambers S and Potvin Kent M, unpublished).

At the national level in Canada, food marketing to children is self-regulated by the food and beverage industry. The voluntary Canadian Children’s Food and Beverage Advertising Initiative (CAI) was implemented in 2007 by 16 large food and beverage companies [26]. Of the 18 companies currently participating, eleven have pledged to refrain from marketing food or beverage products directly to children under 12 years, while the remaining companies have pledged to only advertise “healthier” products to this age group [26]. Each company has defined what constitutes marketing to children, and uniform nutrition criteria defining which products can be advertised to children have been in effect since December 2015 [26]. Regarding the primary school setting, CAI participants are required “to adhere to standards established by schools (…) and by school boards” and must “commit to not advertise food and beverage products in schools” with noted exceptions including charitable activities (e.g., fundraising and educational programs), public service messaging, and food product displays [26].

The purpose of this study was to describe the type of food marketing occurring in a diverse sample of schools in Canada and to examine differences in the frequency of various food marketing activities by school type, province, and socio-economic status of the student body. A secondary objective was to examine Principals’ views and knowledge regarding food marketing generally, and within schools.

## Methods

In fall 2015, contact information for all public school boards in four Canadian provinces (British Columbia, Ontario, Quebec, and Nova Scotia) was accessed through the Canadian Schools Database Directory [27] and approval to conduct research was sought from 217 school boards in these provinces. These provinces were selected because we wanted to obtain information from schools where support and implementation of food-related initiatives was varied, and because they constituted the most populous provinces in western, central, and eastern Canada [28]. Ontario and British Columbia have 13.4 and 4.6 million inhabitants, respectively, while Nova-Scotia has a population of 923,000 [28]. In total, 27 school boards agreed to participate; 13 from British Columbia, 12 from Ontario, and 2 from Nova-Scotia. No school boards from Quebec agreed to participate. Their refusal may be attributable to the fact that Quebec has had provincial legislation banning commercial advertising to children since the 1980s [29]. School boards may have considered the study to be non-pertinent given the policy context.

Administrators (e.g., Principals, Vice-Principals) of schools where board approval was granted were sent an email containing a cover letter explaining the goals of the study and a link to complete a web-based, cross-sectional survey in spring 2016. This survey was available in both official languages of Canada (i.e. English and French). Although this was the methodology followed for the majority of school boards, eleven school boards did not permit direct communication with school Principals. In these cases, surveys were sent directly to Principals from their respective school board. Principals were sent a reminder email approximately two weeks after initial contact was made and again one week after the first reminder was sent. The survey was closed one week after the third and final reminder was received. Participants indicated their informed consent to partake in the study by clicking the “I Agree” button. Ethics approval for this study was granted by the University of Ottawa and the University of British Columbia.

A comprehensive survey was developed based on the seven areas of marketing in schools assessed in previous studies including (1) appropriation of space, (2) corporate sponsorship of programs, events or contests, (3) exclusive marketing arrangements, (4) incentive programs, (5) sponsored educational materials, (6) electronic marketing, and (7) fundraising [10, 14]. The survey questions pertaining to food marketing were adapted from the work of Raine [30], Terry-McElrath et al. [17], Molnar et al. [10], and Froese-Germain et al. [14] by the first (MPK) and second (CEV) authors, both of whom have expertise in food marketing. When responding to the survey, participants confirmed their position at the school (e.g. Principal, Vice-Principals). Principals’ views as to whether food marketing in schools is acceptable and their knowledge regarding its impact on children’s dietary behaviors and health were examined by assessing their level of agreement with various statements using a 5-point Likert scale (ranging from strongly agree to strongly disagree). Principals were also asked whether their school board/district and school had verbal or written policies pertaining to food marketing. In addition, the survey included questions on school characteristics such as school type (primary, middle or secondary), province, and socio-economic status of the majority of students (low, average/middle or high, as assessed by the Principal using a multiple-choice question). Data was collected using FluidSurveys (SurveyMonkey, Ottawa, Canada).

### Data analysis

Data was analyzed using Stata 12 (StataCorp LP, College Station, TX, 2011). School-level descriptive statistics including frequencies, medians, and ranges, characterizing the frequency and type of food marketing present within schools were conducted. Chi-square tests were used to assess differences in school marketing activities and policies by school type (primary versus secondary), province (Ontario versus British Columbia), and socio-economic status (low versus middle/high income). Differences in Principals’ views and knowledge regarding food marketing were similarly examined. Nova Scotia was excluded from the province-based comparisons due to a small sample size (*n* = 6 schools). When chi-square test assumptions were not met [31], a Fisher’s exact test was used. Post hoc z-tests with a Bonferroni correction were carried out when appropriate [32]. As suggested by Field [32], phi and Cramer’s V values were reported as a measure of association for any of the statistically significant bivariate analysis findings. The statistical significance level was set at α < 0.05 for all analyses.

## Results

Twenty-seven out of 145 contacted school boards agreed to participate (18.6% response rate overall; 16% for Ontario, 21% for British Columbia and 25% for Nova Scotia). The final sample consisted of 108 primary schools and 46 secondary schools. Most schools were in either British Columbia (58%) or Ontario (38%), frequently in suburban (42%) or rural (40%) areas. The language of instruction at 83% of schools was reported by Principals as English. More than half (61%) of Principals reported that the majority of students attending their school were of average/middle socio-economic status. School size (e.g., number of students enrolled) was reported by Principals as ranging from less than 200 students (*n* = 32 schools) to ≥1000 students (*n* = 12 schools); all schools with an enrollment of ≥1000 students were secondary schools (Table 1).

**Table 1:** Characteristics of primary and secondary schools including province, language of instruction, location of school, socio-economic status of the majority of students, and size of school

	School type		All Schools, *n* = 154 n (%)
	Primary, *n* = 108 n (%)	Secondary, *n* = 46 n (%)	
Province^†^			
British Columbia	65 (60)	25 (54)	90 (58)
Ontario	38 (35)	20 (43)	59 (38)
Nova Scotia	5 (5)	1 (2)	6 (4)
Language of Instruction^†^			
English	89 (82)	38 (83)	128 (83)
French	6 (6)	4 (9)	10 (6)
English and French	13 (12)	4 (89)	17 (11)
Location of School^†^			
Downtown	19 (18)	8 (17)	27 (18)
Suburban	49 (47)	14 (30)	64 (42)
Rural	37 (35)	24 (52)	61 (40)
SES of Majority of Students^†^			
Low	32 (30)	18 (39)	50 (32)
Average/Middle	68 (63)	26 (57)	95 (61)
High	8 (7)	2 (4)	10 (6)
Size of School (# of students enrolled)^†^			
Less than 200	25 (23)	7 (15)	32 (21)
201–599	71 (66)	18 (39)	90 (58)
600–999	12 (11)	9 (20)	21 (14)
1000+	0 (0)	12 (26)	12 (8)
Presence of a school board policy related to food and beverage marketing^†^			
Written policy	68 (64)	27 (59)	96 (62)
Verbal policy	2 (2)	3 (7)	5 (3)
No policy	9 (9)	4 (9)	13 (9)
Did not know	27 (26)	12 (26)	39 (25)
Presence of a school policy related to food and beverage marketing^†^			
Written policy	32 (30)	16 (36)	48 (31)
Verbal policy	21 (19)	10 (22)	32 (21)
No policy	47 (44)	14 (31)	61 (40)
Did not know	8 (8)	5 (11)	13 (8)

^†^Percentages may not add up to 100 due to rounding

Two thirds of Principals (65%) reported that their school board had a policy (most of which were written) related to the marketing activities of food companies or industry associations in their school. However, 25% of Principals stated that they didn’t know whether their school board had a policy, while 9% indicated that their school board did not have one. Regarding school-level policies, 52% of Principals indicated that their school had a verbal or written policy pertaining to the marketing activities of food companies or industry associations, 40% of Principals reported that their school did not have a policy, and 8% stated that they didn’t know whether their school had one. A significantly larger proportion of Principals from Ontario reported the existence of a written or verbal school-level policy regarding food marketing compared to British Columbia (70 and 46%, respectively; χ^2^ (1, *N* = 136) = 8.13, *p* = 0.005, phi = .245). There were no other statistically significant differences with respect to school board- or school-level food marketing policies by school type, province, or socio-economic status (data not shown).

### Frequency and type of food marketing

Overall, 84% of Principals reported at least one type of food marketing in their school. The median number of distinct types of food marketing per school was 1 for all schools (range 0–6) and for primary schools (range 0–5), and was 2 (range 0–6) for secondary schools. Several types of food marketing were reported by school Principals, the most common of which was schools selling branded food products (e.g., fast food chain restaurants selling pizza or submarine sandwiches, chocolate manufacturers), either directly or through order forms (*n* = 95 schools) for fundraising purposes. The proportion of primary schools selling branded food items was significantly higher (χ^2^ (1, *n* = 148) = 10.80, *p* = 0.001, phi = −.270) compared to secondary schools (Table 2). Of schools that engaged in this type of fundraising, 95% sold either branded pizza (60%), chocolate (52%), or other restaurant/fast food items (43%). Selling branded pizza (67%; χ^2^ (1, *n* = 90) = 8.17, *p* = 0.004, phi = −.301) and other restaurant/fast food items (49%; χ^2^ (1, n = 90) = 5.63, *p* = 0.018, phi = −.250) were each significantly more prevalent among primary schools engaged in fundraising compared to secondary schools (29 and 18%, respectively). By province, a higher proportion of schools engaged in fundraising in British Columbia (69%) sold branded chocolate compared to schools in Ontario (27%) (χ ^2^ (1, *n* = 91) = 15.14, *p* < 0.001, phi = −.408), whereas the proportion of schools selling branded pizza (χ^2^ (1, *n* = 91) = 4.10, *p* = 0.043, phi = .212) and dairy products (χ^2^ (1, *n* = 91) = 7.98, *p* = 0.007, phi = .296) was each significantly higher in Ontario schools (73 and 19%, respectively) as compared to schools in British Columbia (52 and 2%, respectively).

**Table 2:** Frequency and proportion of schools with select types of food and beverage marketing

		School Type		
Type of Food and Beverage Marketing	All Schools, *n* = 156 n (% of schools)	Primary, n = 108 n (% of schools)	Secondary, n = 46 n (% of schools)	*p*-Value^†^
Food or beverage advertisements on property	40 (26)	14 (13)	26 (56)	< 0.001
Food or beverage displays on property	10 (7)	2 (2)	8 (19)	0.001
Exclusive marketing arrangement	23 (16)	5 (5)	18 (45)	< 0.001
Participate in reward or incentive program	27 (18)	26 (25)	1 (2)	0.002
Distribute free branded items	16 (11)	13 (12)	3 (7)	0.558
Fundraise by selling branded items	95 (64)	76 (72)	18 (43)	0.001
Involved in sponsored competitions or contests	3 (2)	2 (2)	1 (1)	1.000
Received money in return for publicity	23 (16)	17 (17)	6 (15)	0.782
Have sponsored programs	5 (3)	4 (4)	1 (2)	1.000
Taken part in market research activities	1 (0.7)	0 (0)	1 (3)	0.278
Promoted/participated in scholarship programs	4 (3)	1 (1)	3 (8)	0.064
Posted student made marketing materials	16 (11)	10 (10)	5 (13)	0.558
At least one type of marketing	129 (84)	92 (85)	36 (80)	0.429

^†^Differences in the type of food and beverage marketing were compared between primary and secondary schools using the chi-square test (*p* < 0.05 was used to determine statistical significance). When chi-square test assumptions were not met, the Fisher’s exact test was used

Advertisements (e.g., posters, signs, equipment) provided by, promoting, or representing a food or beverage company/brand or industry association on school property were reported by 26% of Principals (*n* = 40 schools). Companies or organizations cited as advertising on school property included two large beverage companies (*n* = 17 schools) and various dairy companies or associations (*n* = 9 schools). More Principals from secondary schools (*n* = 26) than primary schools (*n* = 14) reported advertisements from food companies (57 and 13%, χ^2^ (1, *n* = 153) = 31.44, *p* < 0.001, phi = .453) (Table 2). Overall, food or beverage advertisements were most frequently reported as being found on vending machines (*n* = 24 schools;16%) or in hallways, classrooms, or cafeterias (n = 14 schools; 9%). Few schools reported advertisements in “other locations”, for example on food coolers (*n* = 8), athletic fields (*n* = 2), teaching materials (*n* = 2), school media (*n* = 2), student supplies (*n* = 1), recycling bins or garbage cans (*n* = 1) or gym equipment (*n* = 1). Only 14 schools (9%) reported advertisements in at least one of these “other locations”. No Principals reported having food advertisements on disposable dishes or on sports uniforms or bags. In secondary schools, Principals were more likely to report food advertisements on vending machines (42%; χ^2^ (1, *n* = 152) = 33.59, *p* < 0.001, phi = .470) and “other locations” (22%; χ^2^ (1, n = 152) = 12.94, *p* = 0.001, phi = .292) compared to primary schools (5 and 4%, respectively). There were no statistically significant differences in the proportion of schools with advertisements found in hallways, classrooms, or cafeterias between primary (8%) and secondary (13%) schools (χ^2^ (1, *n* = 152) = 1.30, *p* = 0.356), nor were there any significant differences in the location of advertisements by province or socio-economic status of most students (data not shown).

Other forms of marketing such as participation in reward or incentive programs and exclusive contracts with food companies were also reported. For instance, 18% of Principals (*n* = 27 schools) reported that their school took part in a reward or incentive program and 16% of Principals (*n* = 23 schools) stated that their school had an exclusive marketing contract with a food or beverage company or reported receiving financial contributions in exchange for publicity or recognition. The proportion of schools participating in reward or incentive programs was higher in primary schools (25% versus 2%) (χ^2^ (1, *n* = 148) = 9.89, *p* = 0.002,phi = −.259), whereas having an exclusive marketing contract was more prevalent in secondary schools (45% versus 5%) (χ^2^ (1, *n* = 145) = 35.14, *p* < 0.001, phi = .492) (Table 2). Principals from schools where most students were of low socio-economic status were more likely to report distributing free branded items (19%; χ^2^ (1, *n* = 149) = 5.07, *p* = 0.024, phi = −.184) compared to schools where most students were of middle or high socio-economic status (7%). There were no other statistically significant differences with respect to exclusive marketing agreements, participation in reward and incentive programs, receiving money in exchange for publicity, and distribution of free branded items by province or socio-economic status of most students (data not shown). The frequency of other forms of marketing such as scholarship programs (*n* = 4 schools), sponsored competitions or contests (*n* = 3 schools), or market research activity (n = 1 school) was very low.

### Principals’ views and knowledge of food marketing

The proportion of Principals who agreed with the following statements: “It is perfectly acceptable to occasionally give students free samples of branded food or beverage products, regardless of nutritional profile” and “It is only fair that food companies/industry associations gain some commercial benefit from providing schools with funds or other resources” were each significantly higher among those from secondary schools compared to those from primary schools (χ^2^ (2, *n* = 144) = 10.18, *p* = 0.006, V = .266 and χ^2^ (2, *n* = 145) = 7.74, *p* = 0.021, V = .231 respectively). There were no statistically significant differences between primary and secondary schools with respect to knowledge of food marketing (Table 3).

**Table 3:** Principals’ views and knowledge of food and beverage marketing overall and by school type

	Agree n (%)			Neutral n (%)			Disagree n (%)			*p*-Value^†^
	Overall	Primary	Secondary	Overall	Primary	Secondary	Overall	Primary	Secondary	
VIEWS										
I have concerns about the educational value of some teaching materials produced by food and beverage companies/industry associations	93 (64)	67 (64)	26 (63)	45 (31)	32 (31)	12 (29)	8 (5)	5 (5)	3 (7)	0.834^‡^
Schools should be an environment that is free from the commercial pressures children face nowadays	116 (79)	84 (81)	31 (76)	23 (16)	16 (15)	7 (17)	7 (5)	4 (4)	3 (7)	0.642^‡^
Exposing students to food/beverage marketing in schools is an acceptable trade-off for funds or resources that may otherwise not be available	27 (18)	19 (18)	7 (17)	40 (27)	26 (25)	14 (34)	79 (54)	59 (57)	20 (49)	0.534
Schools should not be seen to promote a particular food/beverage brand or company	113 (77)	86 (83)	27 (66)	23 (16)	13 (13)	10 (24)	10 (7)	5 (5)	4 (10)	0.088^‡^
It is perfectly acceptable to occasionally give students free samples of branded food or beverage products, regardless of nutritional profile	20 (14)	9 (9)^**a**^	11 (28)^**a**^	21 (14)	13 (13)	7 (18)	104 (72)	82 (79)^b^	22 (55)^b^	0.006
It is only fair that food and beverage companies/industry associations gain some commercial benefit from providing schools with funds or other resources	24 (16)	11 (11)^**a**^	12 (29)^**a**^	40 (27)	30 (29)	10 (24)	82 (56)	63 (61)	19 (46)	0.021
As long as the school benefits, it does not matter if a food/beverage company or industry association has a commercial motive for providing money or other resources	10 (7)	4 (4)	5 (12)	14 (10)	8 (8)	6 (15)	121 (83)	91 (88)	30 (73)	0.065^‡^
Encouraging students to collect token from items with a high fat, salt or sugar content undermines teaching about healthy eating	119 (82)	83 (81)	35 (85)	13 (9)	9 (9)	4 (10)	13 (9)	11 (11)	2 (5)	0.546
To expose a captive audience of school children to commercial food/beverage messages is exploitative/raises ethical concerns	118 (82)	87 (85)	31 (76)	17 (12)	7 (7)^**a**^	9 (22)^**a**^	9 (6)	8 (8)	1 (2)	0.022^‡^
Most teachers in our school evaluate classroom materials for bias or promotional content	117 (81)	80 (78)	36 (89)	17 (12)	15 (15)	2 (5)	11 (8)	8 (8)	3 (7)	0.258^‡^
KNOWLEDGE										
Schemes that involves collecting tokens/vouchers unfairly influence students to buy certain items when given the opportunity, either at school or in the community	119 (83)	84 (82)	34 (83)	12 (8)	7 (7)	5 (12)	13 (9)	11 (11)	2 (5)	0.347^‡^
Schools involvement with some food/beverage companies/industry associations may have an undesirable effect on the food choices that students make	118 (81)	86 (84)	32 (78)	16 (11)	9 (9)	6 (15)	11 (8)	8 (8)	3 (7)	0.579^‡^
Food and beverage marketing is thought to be associated with childhood obesity	110 (76)	79 (77)	31 (76)	23 (16)	17 (17)	6 (15)	11 (8)	6 (6)	4 (10)	0.699^‡^

^†^Differences in Principals’ views and knowledge of food and beverage marketing were compared between primary and secondary schools using the chi-square test (p < 0.05 was used to determine statistical significance). Post hoc testing (z-test) with a Bonferroni correction was carried out when appropriate. Matching superscripts within rows denote statistically significant differences (*p* < 0.05). ^‡^Chi-square test result is unreliable as one or more cells have an expected value lower than 5

Principals from Ontario were more likely to agree that “exposing students to food marketing in schools is an acceptable trade-off for funds or resources that may otherwise not be available” compared to Principals from British Columbia (28% versus 11%, χ^2^ (2, *n* = 140) = 7.420, *p* = 0.024, V = .230). There were no other statistically significant differences in Principal’s views or knowledge of food marketing by school type, province or socio-economic status of most students (data not shown).

## Discussion

Overall, food marketing was prevalent among our sample of Canadian schools with most Principals (84%) reporting the presence of one or more types of food marketing within their school. Two-thirds of participating schools reported selling branded food items, particularly pizza, chocolate, and other fast food, for fundraising purposes. A quarter of schools reported food advertisements on school property, which were most often found on vending machines, and about 1 in 6 schools reported participating in rewards programs, having exclusive marketing arrangements with food companies or receiving financial contributions in return for publicity.

Our findings suggest that while present, school-based food marketing seems less pervasive in Canada compared to the United States. In US schools, exclusive marketing agreements, reward and incentive programs, and the distribution of free items such as coupons have been found to be particularly widespread [17, 19, 33]. For instance, national surveys have found that 63% of high school students in the United States attend schools with exclusive marketing agreements [33] and 64% of primary school students attend schools where food coupons are distributed as incentives [17]. Advertising, especially on posters and signs, vending machines, food and beverage display cases, and in school media (e.g., yearbooks), has also been found to be pervasive in some US school districts [34, 35]. These types of food marketing were reported considerably less frequently in our sample.

Our results also differed from the few Canadian studies that have documented school-based food marketing. For instance, Velazquez et al. [20] documented food promotions and advertisements in 100% of secondary schools and 80% of primary schools surveyed in Vancouver while only 25% of schools overall reported such advertising in our study. This difference may in part be attributable to the fact that advertisements were self-reported in our study whereas the promotions in Vancouver schools were identified through direct observation. Thus, it is conceivable that the frequency of food advertising on school property reported in our study is underestimated.

The prevalence of exclusive marketing agreements with food companies was also lower in our study (16%) compared to what has been previously reported in Canada. A national survey on commercialism in Canadian schools conducted in 2004–05 found that 27% of schools had an exclusive marketing arrangement with two large beverage companies [14]. This may suggest a decline in exclusive marketing agreements within schools. Such a decline could be explained by the introduction of policies restricting the sale of certain unhealthy food items, such as regular soft drinks, in many Canadian provinces [23–25].

Differences in the types of food marketing present in primary and secondary schools were also noted. For instance, participation in reward or incentive programs was almost solely reported by primary schools, whereas exclusive marketing agreements and food product displays were more prevalent in secondary schools. These results are consistent with studies conducted in Canada, where marketing has been found to differ by school type [14, 20]. For example, Velazquez et al. [20] found that food promotions were more prevalent overall in secondary schools than primary schools. And, in the national survey on school-based commercialism, the frequency of exclusive marketing agreements was higher in secondary schools (60%) compared to primary schools (10%) [14]. Similarly, in the United States, vending of branded food and beverages and exclusive marketing agreements have been found to be more common in secondary than primary schools [17]. As Terry McElrath et al. [17] suggest, these different marketing approaches are likely tailored to the way younger children and adolescents exercise their purchasing power. For instance, incentive/reward programs may be more common in primary schools because they incentivise purchases made by parents, while exclusive marketing agreements and food displays (indicative of more food vending) are more frequent in secondary schools because adolescents have access to more money and can make direct purchases independently.

Contrary to what has been reported in the Unites States [17, 18] and New Zealand [21], our study found few differences in the types of food marketing reported based on the socio-economic status of the student body. According to our only significant finding, schools where the majority of students were of lower socio-economic status were more likely to report the distribution of free branded items than schools where most students were of higher socio-economic status. It is possible that schools located in poorer neighbourhoods distribute more branded foods through their school nutrition/breakfast programs. Despite asking respondents what type of free items were distributed in their school, the quality of our data did not allow us to explore this question further. The dependence of school nutrition programs on the donation of branded foods and their healthfulness merits further investigation and may have implications regarding policy implementation in schools and buy-in from stakeholders.

Although this study did not expressly examine the nutritional quality of foods marketed in schools, our results, consistent with other studies [19, 36, 37], suggest that unhealthy products are being promoted by way of fundraising. For instance, 95% of schools selling branded food items to raise funds reported selling either chocolate, pizza, and/or other fast-food items. The sale of unhealthy branded food highlights the inadequacy of current provincial legislation that exempt fundraising from their school nutrition policies (as is the case in Ontario) [24]. Consistent with other studies [38, 39], it also highlights the lack of enforcement of these policies. For example, 44% of Principals from British Columbia reported that chocolate was sold for fundraising, despite it being prohibited by the province’s guidelines on the sale of food and beverages in schools [23]. This result also shows the limits of the CAI as participating companies choose to exclude fundraising in their definition of “advertising to children”.

The sale of unhealthy foods within schools raises health concerns because it promotes unhealthy dietary behaviors among Canadian children and youth whose diets often exceed the daily recommended intake for sodium and sugar and are inadequate in fruit and vegetables [40–42]. Furthermore, the promotion of foods inconsistent with the tenets of healthy eating taught in schools creates contradictory messages and conflicts with schools’ mission to educate and promote student well-being [43]. That said, even if a branded food item (e.g., pizza from a large chain restaurant) is compliant with school nutrition standards, it is debatable as to whether the sale of this item should be permitted, particularly if children are exposed to a brand that is largely associated with unhealthy products, as is the case for most fast-food and dine-in restaurants in Canada [44, 45]. The potential for such contradictory messages should be considered when crafting food marketing restrictions in schools.

Although most Principals (> 75%) agreed with statements acknowledging the detrimental effect of food marketing on children’s dietary behaviors and health, it is worth noting that nearly half of all Principals sampled either agreed or expressed a neutral position with a statement affirming that the exposure of children to food marketing is an acceptable trade-off for funds or resources that may otherwise not be available. The need for resources may indeed be a key factor driving school-based food marketing and may be breeding ambivalence among administrators regarding the issue. Addressing budget shortfalls and supporting schools to find alternative funding sources will likely be required to gain the support needed to adopt and implement more comprehensive measures restricting food marketing within schools. In fact, economic constraints including the cost of policy-compliant (i.e., healthy) foods, lack of infrastructure (e.g., kitchen) and human resources, coupled with the reliance on food sales to ensure the viability of school cafeterias have all been cited as impediments to the implementation of school nutrition policies in Canada [46–49].

The presence of food marketing in most schools participating in this study also highlights the failure of self-regulation in protecting Canadian children and adolescents from such marketing. Although we did not examine the compliance/behavior of specific companies participating in the CAI, it is evident that this voluntary commitment does not restrict the full breadth of food and beverage marketing activities such as fundraising, reward/incentive programs, and financial contributions in exchange for publicity that is happening in primary schools in which this commitment applies. The CAI also fails to protect adolescents aged 12 and over attending secondary school who seem particularly exposed to the vending of branded food products and advertisements on school property, among other forms of marketing. In addition to its limited scope, the voluntary nature of this initiative also means that it does not apply to several companies or industry associations such as fast food restaurant chains selling pizza or sandwiches and national or provincial dairy associations among others, that have been identified as carrying out marketing (mostly fundraising) in our sample of schools.

Despite documenting whether schools had a policy pertaining to food marketing, Principals were not asked to provide details about these policies. As a result, this study could not investigate whether the presence of these policies was effective in restricting the food marketing activities to which they applied. Regardless, given the presence of food marketing in most schools, our results suggest that the current patchwork of policies has not been effective at protecting children from food marketing in schools. Given these inadequacies, legislation in this area is clearly needed in Canada. In fact, the House of Commons is expected to pass a bill restricting the marketing of unhealthy foods to children under 13 years in 2019 [50].

### Strengths and limitations

This is the first study to examine the full breadth of food marketing in schools across three Canadian provinces. Our findings however are not representative of all schools in Nova Scotia, British Columbia, and Ontario, given the low response rate. As the occurrence of marketing was self-reported, our results are also susceptible to reporting errors and the extent of food marketing may be underestimated. As such, no inferences can be drawn from comparisons made between the results of this study and that of national studies in the United States or other research in Canada. Furthermore, desirability bias may have influenced the expression of Principals’ views regarding food marketing. This study also excluded digital food advertising that appears on websites used in schools by students. For example, a recent study found that coolmathgames.com, a popular website used in schools, featured more than 5 million food advertisements over a one-year period, most of which promoted unhealthy processed foods that were high in either sugar, fat and/or sodium [51]. In addition, the small sample of schools included here did not permit school type or socio-economic status sub-analyses by provinces which constitute different policy environments. Notwithstanding these limitations, this study provided valuable insights regarding the effectiveness of current policies on restricting food marketing in schools which to date has been seldom studied in Canada. This study also provided baseline data on school-based food marketing and did so by examining an exhaustive list of marketing activities and sampling schools from multiple provinces.

## Conclusions

The presence of food and beverage marketing in a high percentage of participating schools highlights the inadequacy of industry self-regulatory policies and the current patchwork of food and beverage marketing policies in place in Canadian schools and school boards. Comprehensive restrictions should be mandated by government in both primary and secondary schools to protect the health of children and youth.

## Abbreviation

CAI: Canadian Food and Beverage Advertising Initiative
